# Analysis of the Psychological Barriers to Spoken English From Big Data and Cross-Cultural Perspectives

**DOI:** 10.3389/fpsyg.2022.899101

**Published:** 2022-06-30

**Authors:** Wen Mao

**Affiliations:** School of Foreign Studies, Nantong University, Nantong, China

**Keywords:** cross-cultural awareness, big data in education, big data mining, spoken English disorder, psychological barrier

## Abstract

College English teaching aims to cultivate students' comprehensive ability to use English. The study of spoken English barriers is a hot topic in this subject area. Based on a survey of non-English primary college students' spoken language impairments, this paper analyzes the research status of spoken language impairments at home and abroad. It relies upon the theoretical basis of Swain's output and Krashen's input hypotheses. With extensive data mining in colleges and universities as the entry point, this paper's content, object, and method are determined by combining qualitative and quantitative research with empirical research. Through the combination of classroom observations, questionnaires, interviews, and other research forms, this paper concludes that the spoken language barriers of non-English primary college students include language and non-language barriers. The influencing factors of the spoken language output barriers include subjective and objective aspects. The questionnaires are analyzed from the three dimensions of the students, schools, and education departments. This paper proposes some ways to overcome the spoken English barriers of non-English college students. It also suggests that non-English college teachers should pay more attention to the cultural transmission of English-speaking countries in English classes, cultivate students' cross-cultural awareness, and enhance students' enthusiasm in English learning. These actions are more conducive to overcoming the psychological barriers in spoken English output.

## Introduction

As informatization rapidly develops, artificial intelligence and big data influence the education industry (Vrasidas, 2000). Today, as the educational scale expands, digitalization, and informatization are constantly promoted and being improved in colleges and universities, causing continual increases in the data volume of scientific research achievements, students' daily behaviors, academic performance, and internet use in colleges and universities (Voogt et al., 2013). Currently, school departments have relatively perfect application systems; each system stores large amounts of student data, but these data are stored only in the database, and no one mines or uses it. Therefore, a current critical issue is how to effectively use a large amount of data through the analysis of data-mining technology to turn raw data into useful information and speed the information construction level of colleges and universities (Voogt and Roblin, 2012).

Translation assumes the existence of two cultures, each of which has its own culture. Thus, it is a double communication activity. To translate correctly, both cultures must be understood (Duan, 2017). Therefore, students' cross-cultural awareness can be cultivated through translation. This ability requires a high standard of spoken English and is the focus of college students in China. This paper analyzes the causes of college students' spoken English barriers and then discovers effective ways of solving these obstacles. Only in this way can the status quo be changed of college students' low spoken communication abilities. College English teachers, relevant education departments, and students must overcome English spoken barriers to improve college students' spoken communication abilities (Huang, 2018).

Many foreign scholars have conducted in-depth research on the factors affecting language learning, such as emotional factors. Bowles (2006) proposed that spoken communication can occur in either a natural or a formal environment. These situations largely depend on the student's type, level, mode, age, and educational level (Bowles, 2006). Bu (2010) believes that multilingual students have linguistic and cognitive advantages and proposes 10 factors affecting second-language mastery. Brantmeier (2013) understands individual differences in emotional regulation through experimental studies and finds that males use automatic emotional regulation more so than females. Johnson (2018) investigated the psychological barriers of Italian–German–English learners from parental educational and language environmental perspectives and found that the parental educational level was a stronger predictor of learners' spoken barriers than was the language environment. Domestic scholars on learners' spoken English barriers are beginning to actively explore the field. Yeme (2007) used the research methods of classroom observations, questionnaires, and interviews to analyze non-English primary college students' spoken English barriers, including vocabulary, thinking mode, and psychological barriers (Chen and Li, 2016). Haiyan (2014) concluded that the barriers to English spoken output are mainly reflected in language and non-language barriers. The language barriers include vocabulary, pronunciation and intonation, and grammar. The non-spoken barriers include topics, psychology, and environment (Zhang and Wang, 2017). In their study, Shenghao and Jinlong (2018) found that most students' spoken language barriers are caused by one of four barriers: psychological, comprehension, phonological, or vocabulary (Xiao and Liu, 2018). Jingjing (2017) believes that the English spoken barriers are as follows: timidity, lack of confidence, nervousness, and anxiety when speaking in English; sparse English vocabulary; poor pronunciation and intonation; little knowledge of British and American cultures; and few occasions for practicing spoken English (Gao and Wang, 2019).

The above literature review indicates that many problems remain in the analysis of the psychological barriers to Chinese students' spoken English output. In particular, psychological issues from the cross-cultural perspective have not yet been examined according to the current research status with the help of research methods in an extensive data analysis of university education. This paper mines the educational data of students' spoken English performance and uses data-mining technology and analysis theory. The findings indicate that students' have psychological barriers to spoken English output. This paper provides scientific theoretical support for the analysis of the psychological barriers to spoken English output from a cross-cultural perspective.

## Two Extensive Data Analyses of Theories Related to Students' Psychological Barriers

### Data-Mining Concepts

**Figure 1:**
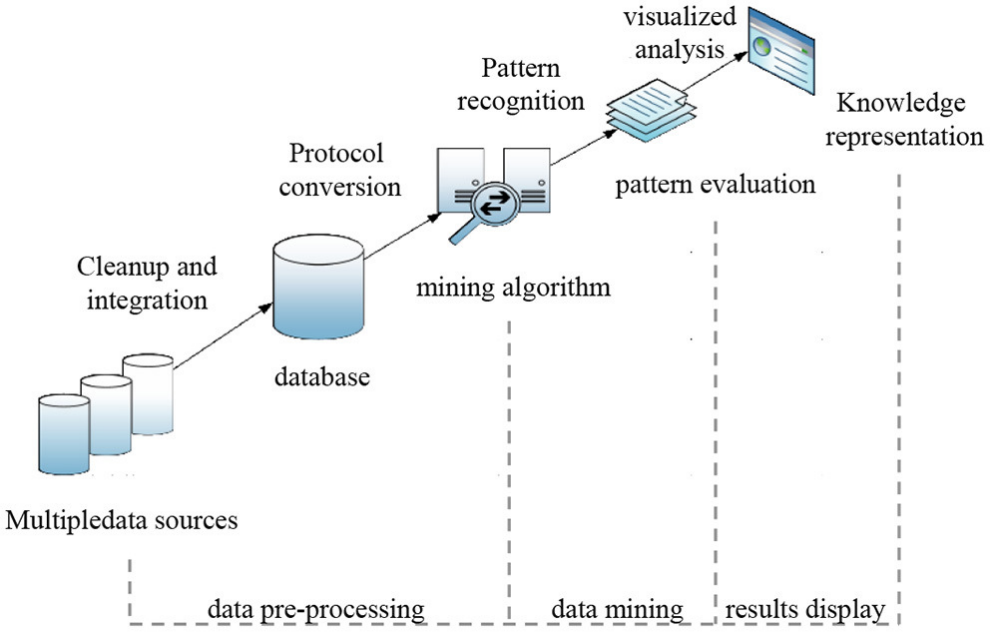
Data-mining process.

The data-mining process is shown in Figure 1. Data mining is generally divided into three stages (Voogt and Pelgrum, 2005): data collection and preprocessing, data analysis and mining, and the evaluation and visualization of results.

The first stage is data collection and preprocessing. It mainly includes collecting the target data, establishing a data warehouse, data cleaning, data integration, establishing protocols, and data transformation (Webb and Cox, 2004). Data collection and preprocessing are the basis and key of data mining, and the data quality determines the effect of the data analysis and mining. Generally, the original data collection includes many invalid data points that need to be culled. By establishing a unified data warehouse and fields, redundant, noisy, and missing data can be deleted or completed with specific rules. Data integration is the unified management of data to facilitate their subsequent use. Data preprocessing is tedious and heavy work, but it also lays a solid foundation for and supports subsequent mining and analysis. The second stage, the data analysis and mining process, is the core of the whole data-mining process. Using the preprocessed data, according to the target of data mining, feature extraction, selection, and model establishment are carried out using specific algorithms. Its algorithms include but are not limited to frequent item set mining, association rules, statistical hypothesis testing, clustering, classification, and regression analyses. The third stage is the evaluation and visualization of the results. Finally, the mining model is evaluated to measure whether the model completes the scheduled tasks and objectives. The data-mining results are analyzed and filtered by specific visualization techniques, and the valuable results are screened and displayed.

### Overview of the Association Rule Methods

Association analysis is one of the more commonly used algorithms in data mining (Venkatesh and Bala, 2008) to discover hidden relations in the features of many datasets. The classic event of an association rule analysis is beer time (Tondeur et al., 2012). A study of sales slips at a department store found that diapers and beer were often sold together, and men bought beer when they bought diapers. Association rules are defined based on frequent itemsets, which are sets of combined items that occur frequently and simultaneously in datasets. When mining the association rules of transactions, support and confidence are the key indicators. To obtain a subitem set shaped like a *B*→*A* relation, the data must satisfy the defined support and confidence degree, where the support degree is the ratio of the frequency of the simultaneous occurrence of A and B (called an itemset) in the dataset to all the itemsets:


$$\begin{array}{l} Support\left(A\to B\right)=\left(AB\right)=\frac{Frep\left(A\cap B\right)}{N} \end{array}$$

Confidence is the proportion of the simultaneous occurrences of A and B to the separate events of A, which can also be understood as conditional probability. The conditional probability of B occurring again under the condition of the occurrence of A is calculated as follows:


$$\begin{array}{l} Confidence=\frac{Frep\left(A\cap B\right)}{Frep\left(A\right)} \end{array}$$

The standard association rules include Apriori and FP-growth. In this paper, the mining association rules of the dataset regarding the students' spoken output anxiety and psychological memory, were based on experience, and the dataset input minimum confidence and minimum confidence are generally divided into two steps:

(1) Screen frequent itemsets. Find frequent itemsets based on minimum support.
(2) Find strong association rule relations in frequent itemsets. These strong correlations satisfy the rules of minimum support and minimum confidence.

This paper uses association rule methods and ideas to mine the psychological data records of students' spoken output anxiety.

### Description of the Hypothesis Testing

Before hypothesis testing, a hypothesis is first proposed. For example, to analyze whether the height distribution of male and female first-year students is different, the following hypothesis is proposed:

(1) The null hypothesis is that male and female students exhibit no differences in their height distribution and is denoted as H_0_.
(2) Alternatively, male and female students exhibit differences in their height distribution, which is denoted as H_1_.

**Figure 2:**
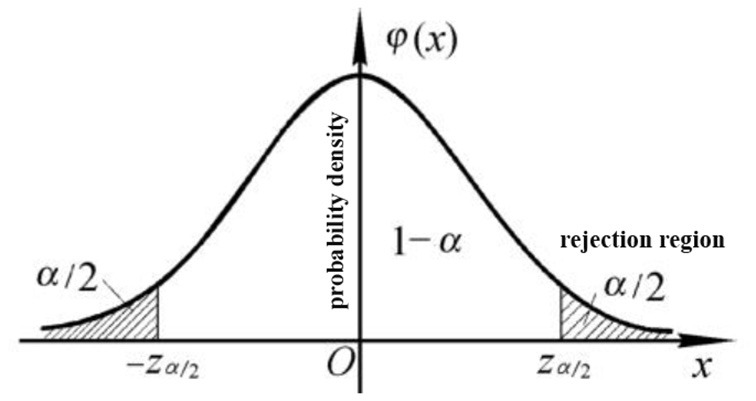
Confidence level and rejection domain in hypothesis testing.

If the male and female students are found to have different height distributions, the null hypothesis is rejected, and the alternative hypothesis is accepted. As shown in Figure 2, in general, a small probability is defined as the rejection domain of a significance level, which is the set of values that reject the null hypothesis test statistics. The set of statistical values that accept the null hypothesis is called the acceptance domain; when the set of rejection fields is defined as M, if *X*∈*M*for random variable A, H0 is rejected. Conversely, if *X*∉*M* H0 is supported, namely:


$$\begin{array}{l} X\in M\Rightarrow \text{refuse }\text{H}0,\text{accept }\text{H}1\\ X\notin M\Rightarrow \text{accept }\text{H}0,\text{refuse }\text{H}1 \end{array}$$

As shown in Figure 2, the boundary dividing the rejection domain is called the threshold.

The significance level is boundary α of the rejection domain, and constants 0.05 and 0.01 are generally used. However, the significance level is not constant, and when α is higher, the null hypothesis is more likely to be rejected. For example, at significance level α = 0.01, when the null hypothesis is accepted, the probability of its correctness is 99%. Therefore, the significance level is the probability of the occurrence of a small probability event in a certain test. The *P*-value in the hypothesis test represents the probability of an event occurring. Here, according to Ronald Fisher (So and Kim, 2009), smaller *P*-values provide greater evidence for rejecting the null hypothesis; that is, the result is more significant. In general, *P*-values are divided into four levels, as shown in Table 1. Significance levels α and P together constitute the evidence measure of whether to reject the null hypothesis. These measures constitute the core of hypothesis testing.

**Table 1:** *P*-value levels.

***P***	**Significance**
<0.01	Very strong evidence to reject *H*_0_
0.01–0.05	Strong evidence to reject *H*_0_
0.05–0.1	Weak evidence to reject *H*_0_
>0.1	Little evidence to reject *H*_0_

For example, the *T*-test (Mostafa et al., 2018) compares the significance of differences between the mean values of two sample types for random data samples subject to a normal distribution. The *T*-test is generally divided into a single- and a double-sample test. As the name implies, a single-sample test means testing whether the population means of the data distribution is acceptable, while a double-sample test requires the same variance of the two samples to test their mean. The *T*-test is a parameter test commonly used in hypothesis testing.

In addition, Wilcoxon's signed-rank test is a non-parametric test in hypothesis testing. It tests whether the distribution of two groups of data is the same when the data do not conform to a normal distribution. Because the data in this paper are different and instable, most of the samples do not conform to a normal distribution, so the Wilcoxon signed-rank test is used to test the distribution difference between the sample populations.

### Data-Mining Classification Model

Classification is an important form of data analysis. The commonly used machine-learning classifiers include logistic regression, naive Bayes (NB), decision tree, k-nearest neighbors (KNN), and support vector machine (SVM).

#### Logistic Regression Classification Model

Logistic regressions are used to obtain a nonlinear model through a linear model transformation for given dataset *N* = (*x*_1_, *y*_1_), (*x*_2_, *y*_2_), …, (*x*_*m*_, *y*_*m*_) to predict as far as possible the actual test value and output it. Although the method is called a regression, it actually addresses the classification model. Compared with other algorithms, logistic regressions are simple in form, explicable, and fast to train. It is mainly applicable to linear classification problems (McFadden, 2015). The main idea is to fit the decision boundary to the greatest extent possible and output the predicted value. Based on the linear regression, that is, for multiple inputs $X={\left(x_{1},x_{2},....x_{n}\right)}^{T}$, the linear expression of predicted output Y is as follows:


$$\begin{array}{l} f\left(x\right)=w_{1}x_{1}+w_{2}x_{2}+\ldots +w_{n}x_{n}+b \end{array}$$

The main idea of a logistic regression is to transform the result of a linear regression into the classification result of a predicted value by function mapping. The sigmoid function is usually selected as the mapping function, and its expression is:


$$\begin{array}{l} g\left(z\right)=\frac{1}{1\text{+}e^{-z}} \end{array}$$

**Figure 3:**
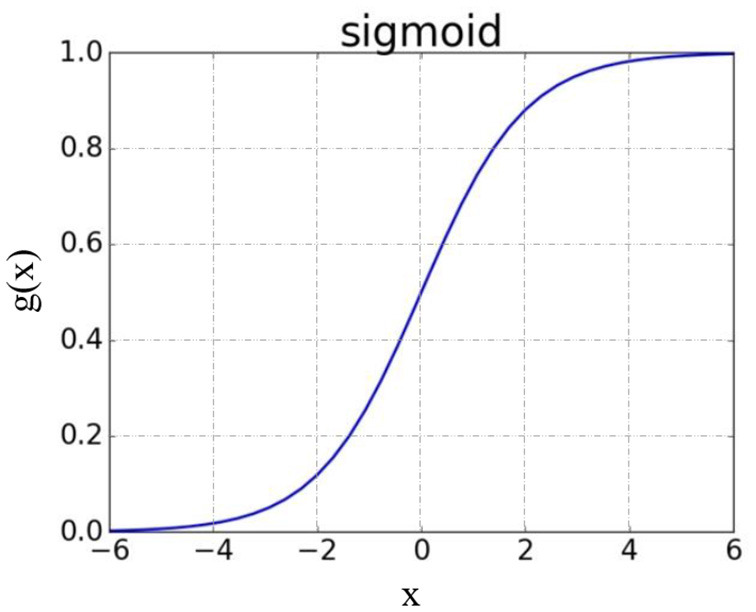
Sigmoid functional image.

As shown in Figure 3, the image is shaped like an S-shaped curve.


$$\begin{array}{l} g\left(z\right)=\frac{1}{1\text{+}e^{-\left(w^{T}x+b\right)}} \end{array}$$

The figure shows that the final value of *g*(*x*) is:


$$g\left(x\right)=\{\begin{array}{l} 1\;w^{T}x+b0\\ 0.5\;w^{T}x+b\text{=}0\\ 0\;w^{T}x+b0 \end{array}$$

Any real number whose domain is R can be mapped between [0, 1] through the sigmoid function, and the sample can be determined as positive or negative by judging whether the value of *g*(*x*) is greater than the reading value of 0.5. Practically, different thresholds can be adjusted according to the actual situation. When solving, a logarithmic transformation is usually performed to obtain:


$$\begin{array}{l} \ln\;\frac{g\left(x\right)}{1-g\left(x\right)}=w^{T}x+b \end{array}$$

where *g*(*x*) is regarded as the probability that *x* is a positive example, and 1−*g*(*x*) is the probability that *x* is a negative example. Therefore, using a logistic regression algorithm to classify problems ultimately transforms into solving optimal *w*. The model's loss function, that is, the difference between the predicted and the actual values, is used to optimize the model classification results. When solving the model parameters, the gradient descent method is used to solve the minimum loss function. The logistic regression classification model is simple to implement, has a small calculation, and is easy to understand and implement. However, when the feature space is large, the classification performance will be affected.

#### Other Classification Models

(1) NB

NB is a marker that considers how to select the optimal category based on probability and misjudgment in a probabilistic framework (Denson et al., 2015). The core idea is to calculate the probability of samples appearing in different categories through Bayes's theorem. This algorithm requires that features be independent of each other. When NB is used, the probability value after the exchange of two conditions is usually obtained after a certain conditional probability is known. Its Bayes's theorem is as follows:


$$\begin{array}{l} P\left(A|B\right)=P\left(B|A\right)P\left(A\right)/P\left(B\right) \end{array}$$

The NB model relies on classical mathematical theory, the algorithm theory is simple, and the classification model is robust although not sensitive to missing data. However, its classification accuracy depends on prior knowledge, so the accuracy will be poor due to the prior model.

(2) KNN

No explicit training process is needed for the KNN classification algorithm (Li et al., 2016). The main idea of its classification is to find the k data samples closest to a given test sample based on some distance measurement method and to make a prediction based on close information. KNN's classification idea can be understood as the proverb, “birds of a feather flock together.” In actual classification, for sample data across regions or with large sample overlaps, the KNN classification effect is better, and KNN can be used for nonlinear classifications. However, KNN requires a large computation amount and results in low classification accuracy for a few sample types when the sample data are unbalanced.

(3) SVM

SVM (38) aims to find *N* = (*x*_1_, *y*_1_), (*x*_2_, *y*_2_), …, (*x*_*m*_, *y*_*m*_), which is the partition hyperplane in the sample space for dataset A and which can be described by Formula (1), where *w* is the normal vector, which determines the direction of the partition plane, and b is the displacement. Its objective is to solve the parameters, continually optimize the model process, and then transform to solve the convex quadratic programming problem, which minimizes the loss function through regularization to improve the accuracy of the model classification.


(1) $$\begin{array}{r} \vec{w}\cdot x+b=0 \end{array}$$

**Figure 4:**
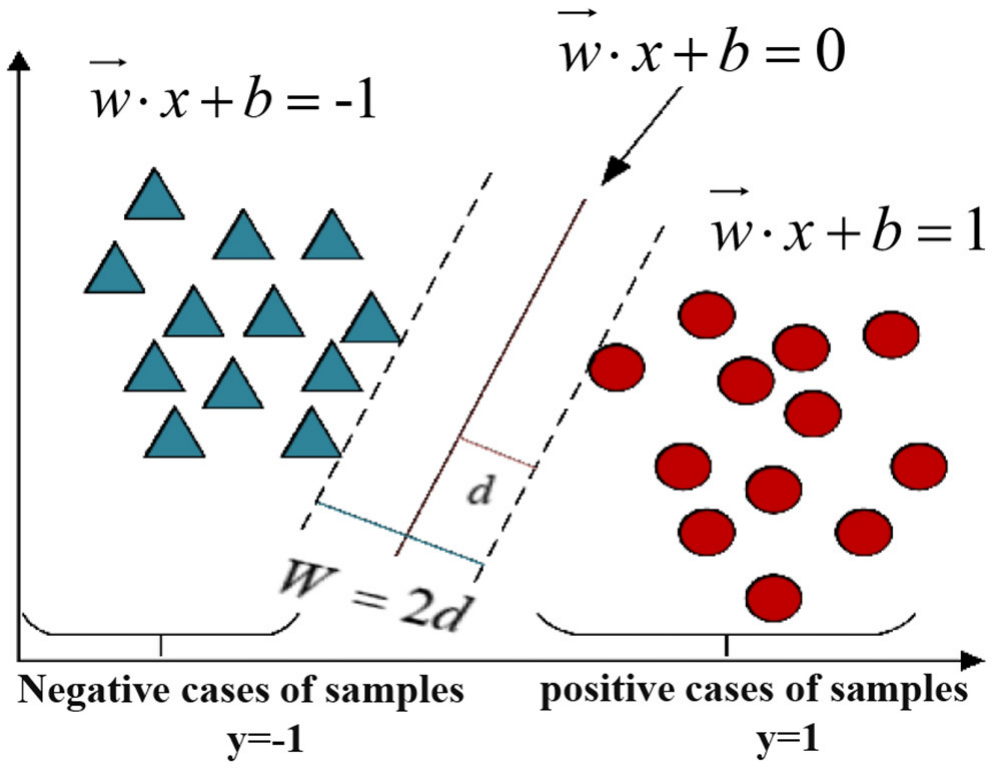
SVM classification model diagram.

Figure 4 shows the SVM classification model diagram.

As shown in Figure 4, the core idea of SVM is to separate the positive and negative samples through this plane. The SVM algorithm has strict theoretical support and does not rely on statistical methods. The complexity of its calculation lies in the number of sample support vectors, which is not affected by feature dimension. It is suitable for high-dimension data samples but not for large-scale samples (Jiang).

(4) Decision tree

The decision tree classification model is used to train the sample data based on the known probability of all possible occurrences and calculates the distribution of each expected value according to the minimization principle of the loss function of the built number. Its essence is to select a feature value with the greatest benefit for dividing the tree using the idea of “divide and conquer.” With continual division, the samples contained in one branch of the tree belong to the same category (Tang, 2016). The general classification steps of a decision tree are sample feature selection, spanning tree, and tree pruning (optimization). Commonly used decision tree generation strategies include Iterative Dichotomiser 3 or C4.5.

The decision tree classification algorithm is highly interpretable, and the importance of features can be seen clearly through the spanning tree, so it is suitable for high-dimensional data. However, the correlation between feature attributes is easy to ignore and the method is greatly affected by the data samples, which are usually prone to over-fitting and not sufficiently robust.

## Research Design

### Data Acquisition and Preprocessing

The school business division is clear; each department has its own independent mature business system, such as the logistics, educational administration, and library systems. Each system stores a large amount of student data, but their storage format is not uniform, and some of the data are dirty and redundant. Therefore, before analyzing the student data, this paper preprocesses them, including data cleaning and desensitization as well as unified formatting storage. The purpose is to facilitate the analysis and research of the student behavior data in the subsequent sections.

### Data Collection and Description

To make a more comprehensive comparative analysis of students' behaviors, this study used the years 2018–2020. This sample of undergraduate students enrolled in March. First, these years include three undergraduate grades, namely, 2018, 2019, and 2020. By 2021, the spoken output of the desensitization data of the students with psychological abnormalities numbered 57. Such students had obvious serious psychological abnormalities in their spoken lessons. By interviewing their teachers and analyzing the interview results, it is found that such students have poor psychological states that are their teachers' focus, which is the main object of this paper. The students with psychological abnormalities in spoken English class were associated with the school student database, and their basic information was extracted, including gender, ethnicity, class, and major. On this basis, 1,733 students with a normal performance were used in this experiment. The students with a normal performance included all the students in the class of the students with an abnormal performance. If the class of student A with an abnormal psychology was 2018 Communication Engineering Class 2, all the students in this class were added to the list of students with a normal psychology. In this way, the other students in the class of the 57 students with spoken English impairments formed the list of the students with a normal performance. This experiment used 1,790 students. The basic information of the students is shown in Table 2 (the data have been desensitized).

**Table 2:** Students' basic information.

**Student ID**	**Gender**	**National**	**Faculty professional**	**State of mind**
1001	Male	Han	2018-1	Normal
1002	Female	Han	2018-1	Normal
……	……	……	……	……
1790	Male	Han	2020-400	Normal

### Data Preprocessing

The campus database studied in this paper contains a large amount of student privacy data, especially student psychological conditions that, once leaked, may add to the psychological burden of students. While using these data to research, the authors of this paper also have obligations and responsibilities. Student privacy must be protected. Under the circumstance of massive student data, how to protect students' privacy is also a primary concern of this paper.

In this paper, student numbers are associated with the checking of the students' performance records and other campus data. After the data were obtained, the student numbers were desensitized. Certain rules were used to transform the student identification data, thus hiding the real student identification information. In the process of the data use, the students' names were deleted, and the student number after desensitization was used as the unique identification. In addition to using the student numbers, this paper also replaces and desensitizes the students' identifying information in space and time. Without destroying the original nature of the data, it greatly protects student privacy. In addition, none of the data used in this article may be disclosed to third parties or used for any commercial purposes.

#### Preprocessing

The following were the problems found in the data obtained in this experiment and their solutions:

(1) The data format was not unified

Because the data in this article come from different systems, the data fields and storage formats are different. Therefore, this paper used data integration technology to store heterogeneous data in a unified way in a database. In addition, multiple tables were associated with the desensitized student number as the main key, and one-hot encoding (Tang, 2016) was used to replace gender, ethnicity, and other characteristics.

(2) Data were missing

For the missing data in this paper, the following principles were followed: (i) Ignore tuples. If the amount of data was sufficiently large and part of the student's data was missing, either this data or the current student's information was deleted. If a student was missing scores from a large number of courses, that student was removed from the student list in the scores module. (ii) Fill in data according to the data distribution. For example, in the academic performance data, if a student had missing scores for an individual course, the average score for this semester was used to fill in the data.

(3) The data were noisy

Some of the data exported from the database had errors or outliers that deviated too greatly from the other data. For example, a student's total score of 999 in a single subject is noisy data because the school adopts the percentage system. However, another kind of outlier was not dealt with here. For example, consider the case of a student who fails to pass exams in 15 subjects. Although this data point deviates from the data center, this is the actual data, which is also the data of the students concerned in this paper. Thus, the noisy data were mainly divided into two categories: abnormal data that cannot be factual and are usually the result of interference, which were deleted, and real abnormal data, which are a focus of this experiment. Therefore, some of the data can be visualized, and the noisy data can be eliminated according to the specific situation.

(4) Some of the data were redundant

Some redundant data and fields were deleted. For example, the teacher information in the score data is not the object of this study, so it was deleted. After the above preprocessing steps were completed, the relevant data table is finally obtained of the students in the school and the dataset size. The dataset here contains all the data generated during the period of the students' schooling through June 2021, namely, 4-year schooling data of students for grade 2018, 3-year schooling data of students for grade 2019, and 2-year schooling data of students for grade 2020. In the subsequent experiments, the data from these periods are screened according to the actual needs.

#### Research Methods

The research methods used in this paper include classroom observations, questionnaire surveys, and interviews.

(1) Classroom observations

The classroom observation table used in this study is divided into three parts: teacher, student, and interaction. In the classroom observations, the author mainly observed the teachers' teaching contents, processes, methods, language used, and activities. The author also observed the students' spoken English status, basic language situation (including pronunciation and intonation, grammar, and vocabulary), the English-speaking state, and classroom participation. In terms of interactions, this study focuses on the methods, language, and evaluations of the interactions between the teachers and the students. The classroom observations supplement the questionnaire surveys so the causes of the college students' spoken English barriers can be summarized more comprehensively and convincingly and corresponding countermeasures can be suggested.

(2) Questionnaire surveys

Questionnaires were administered to the 1,790 student samples selected from the aforementioned big dataset. According to the scoring content and standard of the college English test (CET-4 and CET-6; speaking portion), combined with the dimension of the English hearing impairment questionnaire, the two dimensions of spoken English impairment (language and non-language impairments) were ultimately determined. According to the study purpose, questions with low correlations were removed, and the study questionnaire was ultimately formed after repeated modifications.

The questionnaire mainly consists of two parts with 40 questions. The first part (Questions 1–5) is intended to understand the students' basic information and current situation of spoken English, such as gender, grade, major type, family location, etc. The second part (Questions 6–40) investigates the students' spoken English barriers and their causes. Questions 6–9 are about pronunciation and intonation barriers in spoken English. Question 10 is about grammatical barriers. Questions 11–12 are about vocabulary problems. Questions 13, 14, 17, and 20–22 are about psychological barriers. Questions 23–30 are about learning methods and habits. Questions 15, 16, and 31 are about cultural barriers. Questions 18, 19, 32–40 are about environmental barriers. The questionnaire employs a Likert-type five-level scale, and the students chose the answers according to their actual situation. In addition, the questionnaire reliability was analyzed by SPSS 23.0 statistical analysis software, and the questionnaire reliability coefficient (Cronbach alpha) used in this study was 0.869, which was higher than 0.7, indicating that the questionnaire was reliable. Table 3 shows the categories of spoken English barriers.

**Table 3:** Spoken English barrier categories.

**Spoken English barrier**	**Sub-classification**	**Question numbers**
Language barriers	Phonological and intonation barriers	6, 7, 8, 9
	Grammatical barriers	10
	Vocabulary barriers	11, 12
Non-language barriers	Mental barriers	13, 14, 17, 20, 21, 22
	Learning methods and habits barriers	23–30
		15, 16, 31
	Environmental barriers	18, 19, 32–40

(3) Interviews

According to the questionnaire results, interviews were further conducted of the teachers and students. The first interview was conducted with eight non-English majors, from freshmen to seniors. One male and one female from each grade were randomly selected as the interviewees. The interview results showed that most of the non-English major college students thought their English spoken barrier were mainly due to personal reasons, and other objective factors had a great impact on their English spoken barriers.

The second interview was mainly aimed at teachers. Eight teachers participated in the interview, among whom four teachers were interviewed by telephone. Most of the interviewed teachers believed that the main reason for the students' spoken English difficulties was their poor language foundation and psychological barriers. At the same time, many suggestions were made for the relevant education departments and college English education.

## Results and Discussion

### Analysis of the Questionnaire Survey Results

SPSS 23.0 software was used to analyze the collected questionnaires, and the first research question was, “What are the spoken English barriers of non-English major college students?” Two commonly used measures [means and standard deviations (SD)] were used for the analysis. Table 4 presents the descriptions and statistics of the non-English major college students' oral English barriers, including the means and SDs of the seven types of English spoken barriers identified.

**Table 4:** Spoken English barriers.

**Spoken English barrier**	**Sub-classification**	**Mean**	**SD**	**Amount**
Language barriers	Phonological and intonation barriers	6, 7, 8, 9	3.50	292
	Grammatical barriers	10	3.56	292
	Vocabulary barriers	11, 12	3.76	292
Non-language barriers	Mental barriers	13, 14, 17, 20, 21, 22	3.12	292
	Learning methods and habits barriers	23–30	2.88	292
	Cultural barriers	15, 16, 31	3.11	292
	Environmental barriers	18, 19, 32-40	2.27	292
Total			3.29	292

The results in Table 4 show that the average sample value was 3.29 (SD: 0.90), indicating that the non-English major college students have spoken English difficulties. In descending order, the mean values of the spoken language barriers were vocabulary (mean: 3.76; SD: 0.85), grammar (mean: 3.56; SD: 0.94), voice and intonation (mean: 3.50; SD: 0.87), and psychological barriers (mean: 3.12; SD: 0.92); those of the non-language barriers were cultural (mean: 3.11; SD: 0.91), learning methods and habits (mean: 2.88; SD: 0.92), and environmental barriers (mean: 2.72; SD: 0.97). Among them, the vocabulary barrier was the highest, and the environmental barrier was the lowest.

The averages of the seven barrier types were between 2.5 and 3.4, which belonged to the middle-frequency range, according to Oxford's classification. The mean of the vocabulary barrier was the highest, indicating that vocabulary was the biggest problem for the non-English major college students when speaking English. Among the seven barrier types, the environmental barrier had the lowest average value, indicating that the non-English major college students' spoken English was less affected by the environment. The standard deviations were all relatively low, below 1.00, indicating that the students had similar attitudes toward the seven subcategories.

(1) Language barrier

Table 5 shows the statistics on the language barriers, including the pronunciation and intonation, grammar, and vocabulary barriers.

**Table 5:** Language barriers statistics.

**Language barrier**	**Question number**	**Mean**	**SD**
Phonological and intonation	6	3.58	0.87
	7	3.57	0.81
	8	3.45	0.86
	9	3.40	0.93
Grammatical	10	3.56	0.94
Vocabulary	11	3.92	0.83
	12	3.58	0.92

The average value of Question 6 (“I can't pronounce the International Phonetic Alphabet correctly, which affects my spoken expression”) was 3.58 (SD: 0.87), and that of Question 7 (“I can't pronounce every word correctly, which affects my spoken expression”) was 3.57 (SD: 0.81). The average value of Question 8 (“I can't distinguish the different meanings of the same sentence with different intonations, which affects my spoken expression”) was 3.45 (SD: 0.86), and that of Question 9 (“When speaking English, I don't know the appropriate intonation to use to express my ideas”) was 3.4 (SD: 0.93). Among these questions, Questions 6 (mean: 3.58; SD: 0.87) and 7 (mean: 3.57; SD: 0.81) had higher scores, indicating that the non-English major college students had difficulties with English phonetic symbols and word pronunciation. Question 10 (mean: 3.56; SD: 0.94) regarded the grammatical barriers. Questions 11 (mean: 3.92; SD: 0.83) and 12 (mean: 3.58; SD: 0.92) were about lexical barriers. These questions were classified as lexical barriers because the author learned from the classroom observations and interviews with the teachers and the students that the non-English major college students had poor listening abilities because they did not understand the vocabulary in the listening passages. Compared with the pronunciation and intonation as well as the grammar barriers, the mean value of the vocabulary barriers was higher (in Question 11, 22.95% of people chose “general,” 48.97% chose “agree,” and 24.32% chose “completely agree”), indicating that the vocabulary barriers were the most obvious language barriers.

(2) Non-language barriers

The non-language barriers included learning methods and habits and cultural and environmental barriers. As shown in Table 6, six questions were asked about psychological barriers, including about anxiety, confidence, interest, and motivation, etc.

**Table 6:** Non-language barrier statistics.

**Non-language barriers**	**Question number**	**Mean**	**SD**
Mental	13	3.52	0.94
	14	3.57	0.96
	17	3.82	0.84
	20	2.45	1.01
	21	1.98	0.97
	22	3.68	1.08
Learning methods and habits	23	2.60	1.00
	24	2.76	0.99
	25	3.27	0.94
	26	3.04	0.95
	27	3.32	0.89
	28	2.60	0.97
	29	2.83	0.97
	30	2.66	1.01
Cultural	15	3.34	0.91
	16	3.34	1.02
	31	2.55	0.91
Environmental	18	3.73	0.96
	19	2.89	1.09
	32	2.03	0.89
	33	2.24	0.92
	34	2.10	1.02
	35	1.99	1.08
	36	2.78	1.01
	37	2.62	0.95
	38	3.15	1.10
	39	2.73	0.97
	40	3.15	1.0

The mean value of Question 13 (“I feel anxious and nervous when speaking in English, which affects my spoken expression”) was 3.52 (SD: 0.94). The mean value of Question 14 was 3.57 (SD: 0.96). The average value of Question 17 (“My enthusiasm for spoken practice is not high, and I seldom speak in class and after class, which affects my spoken expression”) was 3.82 (SD: 0.84). The average value of the three questions was higher than 3.5, which was in the high-frequency range, indicating that the students had strong psychological barriers such as anxiety and confidence. The mean value of Question 20 (“I am not interested in learning spoken English”) was 2.45 (SD: 1.01). The mean of Question 21 (“I think speaking fluent English is not important for learning English”) was 1.98 (SD: 0.97). The mean value of Question 22 (“My purpose in learning English was not only to pass the CET-4 and CET-6”) was 3.68 (SD: 1.08), indicating that most of the students were interested in speaking English fluently and had different motivations. In summary, the teachers should continue to stimulate and maintain their learning motivation and interest in English learning in their daily teaching while trying their best to help the students overcome adverse effects such as anxiety and low confidence.

Questions 23–30 were related to learning methods and habits. Table 6 shows that the average values of the 8 questions were between 2.5 and 3.4, belonging to the medium-frequency range, indicating that the learning methods and habits of the non-English major college students had an impact on their spoken English impairment. In addition, the mean value of Question 25 (“I do not want to practice spoken English with classmates or alone after class”) was 3.27 (SD: 0.94). Question 26 (“I don't want to use my spare time reading English-related books or newspapers”) had an average of 3.04 (SD: 0.95). This result, combined with the analyzed psychological barriers, suggested that the students had strong motivation toward and interest in spoken English, but their subjective initiative was not strong, which was an important factor causing the spoken language barriers. The mean value of Question 27 (“I spend less time practicing spoken English than other English-learning skills”) was 3.32 (SD: 0.89), which indicated that the students did not attach great importance to spoken English. The teachers should help students attach great importance to spoken English subtly through their daily teaching.

Questions 15, 16, and 31 were related to the cultural barriers. Question 15 (“My lack of understanding of the cultural differences between the East and the West affects my spoken expression”) had the highest mean (mean: 3.34; SD: 0.91), indicating that in spoken communication, due to the differences in cultural background knowledge, the non-English major college students had spoken difficulties. In addition, according to Question 16 (“When speaking English, I cannot eliminate the influence of the difference in thinking mode between my mother tongue and English, which affects my spoken expression”), the value (mean: 3.44; SD: 1.02) indicated that the mother tongue had a profound influence on the students' spoken English.

Questions 18, 19, and 32–40 were related to the environmental barriers, which involve the teachers, classroom environment, and campus and family environment. The average value of Question 18 was 3.73 (SD: 0.96). The average value of Question 38 (“The radio station at my school does not often broadcast English-related programs) was 3.15 (SD: 1.10), which indicated that the English spoken barrier of the non-English major college students was closely related to their language environment.

The non-English major college students had two barriers to spoken English: language and non-language. When the two were compared, the average language barrier was higher, indicating that the language barrier was more obvious. The language barriers included phonological and intonation, grammatical, and lexical barriers. The average of the three barriers in descending order was lexical > grammatical > phonological and intonation barriers. The non-language barriers included psychological, learning methods and habits, and cultural and environmental barriers. The average of the three barriers in descending order was psychological > cultural > learning methods and habits > environmental barriers. The average value of the psychological barriers was the highest, indicating that the psychological factors had the strongest influence on the spoken English of the non-English major college students. However, the learning methods and habits, cultural, and environmental barriers also affected the spoken English barriers of the non-English major college students.

### Analysis of the Classroom Observation Results

The author started classroom observations and video recordings on September 17, 2021. The author mainly observed the students and teachers from the two aspects of language and non-language. Regarding the students, the author mainly focused on their pronunciation and intonation, grammar, vocabulary, English-speaking status, and classroom participation. For the teachers, the author mainly focused on their teaching content, process, methods, activities, and language. In addition, the interactions between the students and the teachers were an important part of these class observations. After observing 20 English-listening and English-speaking classes in four freshmen and sophomore classes of non-English majors, the author made the following findings:

First, the author summarized the teacher level as follows: (1) Teaching content—The teachers mainly taught the textbook contents, with frequent interactions between the teachers and the students. (2) Teaching process—The teaching process of the four teachers was very clear, and each teacher attached great importance to course introductions and stimulated the students' interest in learning. (3) Teaching activities—Spoken class activities were conducted in a single form, with only two discussion groups. (4) Teaching language—The teachers did not use English well in class. Only one English teacher taught almost entirely in English. One English teacher taught mainly in English and Chinese, and two English teachers taught in both Chinese and English. (5) Teaching media—The four English teachers all used multimedia and other electronic devices, giving full play to the benefits of modern educational technology. The teaching content displayed on the screen was elaborate and vivid, which was beneficial for motivating the students to learn. (6) Classroom participation—Only a small number of the students actively raised their hands to speak or participated in class activities. Most of the students did not participate enthusiastically in class and spoke English only when they were addressed directly by the teacher. Some of the students were silent during the entire class, and they neither spoke nor participated in class activities such as group discussions. (7) Homework arrangement—The four English teachers assigned little spoken English homework.

Second, the author summarized the student-level content observed in class. (1) Students' spoken English in class—The students had a weak language foundation. Only a few of them could answer questions fluently, correctly, or vividly, and most of them needed their teachers' help to state their views clearly. (2) Pronunciation and intonation—Most of the non-English majors had good pronunciation and intonation, but a few did not have standard pronunciation. (3) Vocabulary—The students did not have a strong vocabulary. The majority of the students hesitated or paused when expressing their ideas in English, and sometimes they needed their teachers' help expressing their ideas smoothly. (4) Grammar—The students made many grammar errors, especially with verb tenses. (5) Speaking English—Due to the serious psychological influence of anxiety, shyness, and lack of confidence, most of the non-English major college students did not actively participate in spoken exercises and did not answer questions confidently. (6) Practice—The students had few opportunities to practice spoken English.

In summary, the author preliminarily finds that the non-English major college students have difficulties in spoken English pronunciation and in intonation, grammar, vocabulary, psychology, and other aspects. Therefore, the author analyzed the causes of these specific obstacles.

### Analysis of the Interview Results

Interviews were conducted with the students and teachers in December 2018. First, the author randomly selected 4 from 292 students for an interview, namely 2 boys and 2 girls. Then three non-English majors were interviewed. Each interviewee was interviewed in Chinese for ~20 min. Before the interview, the author clarified the purpose and confidentiality of the interview and recorded the whole process with a recording pen with their permission.

(1) Analysis of the student interview results

These interviews had four questions: First, “what obstacles do you think you have in speaking English?” Second, “what do you think are the causes of these obstacles?” Third, “does your mother tongue affect your spoken English?” Finally, “do you have any suggestions for improving students' spoken English?” The four students all agreed that the biggest spoken language barrier was vocabulary. One boy thought he had a strong grammar barrier and did not know how to express his ideas using English grammar. One girl thought she had a large phonetic barrier because she did not learn phonetic symbols in primary school, so she had to use Chinese pinyin to mark the new words she learned. They believed that there were two reasons for the emergence of the obstacles, subjective and objective, but they were more deeply influenced by the subjective factors. These included the students' weak language foundation, insufficient attention given to spoken English, low motivation in learning spoken English, adverse psychological barriers (anxiety, shyness, and inferiority), and a lack of understanding of the culture of English-speaking countries. The objective reasons included too many students in classes, few opportunities to practice spoken English in class, a lack of understanding of Western cultural background knowledge, few activities provided by the school to practice spoken English, and the teachers' traditional teaching methods. The students felt that their mother tongue had no effect on learning English. They hoped that the school could establish more activities such as English corner and English square and give bonus points to the students who participate in the comprehensive test.

(2) Analysis of the teacher interview results

These interviews were conducted around four questions: First, “what barriers do you think non-English major college students have in their spoken English?” Second, “what do you think are the causes of these spoken English barriers?” Third, “do you think the mother tongue has any influence on students' spoken English abilities?” Finally, “do you have any suggestions for improving students' spoken English?” The three teachers all believed that the non-English major college students' spoken English barriers mainly manifested in two aspects: vocabulary and psychology. The obstacles were caused, on the one hand, by the students, who did not realize the importance of spoken English, did not pay attention to vocabulary accumulation, had low classroom enthusiasm, did not want to speak English in public, and were afraid of speaking incorrectly. On the other hand, because teaching tasks were limited, listening and speaking classes offered students less time to practice spoken English, and class sizes were too large, which was not conducive to the teachers' spoken English teaching. Finally, the three teachers also gave sincere suggestions: First, the school should reduce the number of students in each class, preferably to within 30. Second, the school can hold an English salon to consider cross-cultural factors, which will improve students' interest in spoken English. Third, teachers should help students realize the importance of spoken English in their daily teaching. Finally, students who truly want to improve their spoken English abilities or who rely on their own efforts should take the initiative to find channels and skills to improve.

In summary, after a comprehensive analysis of the classroom observations, questionnaires, and interviews, the author found that the non-English major college students had spoken English barriers in two respects: language and non-language barriers. The language barriers included grammar, pronunciation and intonation, and vocabulary barriers, while the non-language barriers mainly included learning methods and habits, psychological, cultural, and environmental barriers. This study is characterized by the following innovative results: First, the author used two commonly used measurements (means and SDs) to compare all kinds of obstacle values. Second, the size according to the Oxford Frequency Table was used to determine the main impediment for the non-English major college student type to formulate a targeted strategy. The analysis results show that the average value of the language barrier was higher than that of the non-language barrier, indicating that the language barrier was more obvious. The language barriers included phonological and intonation, grammatical, and lexical barriers. The averages of the three barriers ranked in order as follows: lexical > grammatical > phonological and intonation barriers. The non-language barriers included psychological, learning habits and methods, cultural, and environmental barriers. Their average rankings were psychological barriers > cultural > learning methods and habits > environmental barriers. The average value of the psychological barriers was the highest, indicating that these barriers had the strongest influence on the spoken English of the non-English major college students. The results show that we can take the mother tongue culture as the breakthrough point, attach importance to the integration of cross-cultural factors in English teaching, improve students' awareness of the mother tongue culture in English class, and then help non-English majors to overcome spoken English barriers and improve their spoken English spoken levels.

## Conclusion

This paper analyzes the psychological barriers to college students' spoken output by using existing big data on college education. After a massive, cluttered data-cleaning effort, specifications, such as preprocessing and proposing a mining model of students studying their in-school performance, investigation data were extracted for the spoken English barriers of 1,790 freshman, sophomore, and junior college students who were non-English major college students. The questionnaires, classroom observations, and interviews were quantitatively analyzed to discover the manifestations of the spoken barriers. The findings are as follows:

(1) Non-English major college students have spoken English barriers that fall into two main categories: language and non-language barriers. The former are reflected in three barriers: pronunciation and intonation, grammar, and vocabulary. The ranking by the average values is vocabulary > grammar > pronunciation and intonation barriers. The average value of the vocabulary barriers is the highest, and its impact is the most serious. The non-language barriers include psychological, learning methods, learning habits, cultural, and environmental barriers. The ranking by the average values is psychological > cultural > learning methods and habits > environmental barriers. The average value of the psychological barriers is the highest, indicating that the psychological factors have the strongest influence on college students' spoken English output.
(2) Regarding the subjective and objective reasons, the factors affecting non-English major college students' spoken English barriers are identified. The subjective factors mainly include three aspects: (1) Students are affected by their anxiety, vanity, cowardice, and other adverse psychological factors. (2) Students' existing knowledge levels are insufficient. (3) Students have poor learning methods and habits. The objective factors can be summarized into five aspects: (1) Students lack background cultural knowledge. (2) The students' mother tongue interferes with their spoken English learning. (3) The students lack a real language environment. (4) Class capacities are too large. (5) The students are influenced by the teachers' levels, teaching methods, and teaching means.

## Data Availability Statement

The raw data supporting the conclusions of this article will be made available by the authors, without undue reservation.

## Ethics Statement

The studies involving human participants were reviewed and approved by Ethics Committee of Nantong University. Written informed consent for participation was not required for this study in accordance with the national legislation and the institutional requirements.

## Author Contributions

The author confirms being the sole contributor of this work and has approved it for publication.

## Conflict of Interest

The author declares that the research was conducted in the absence of any commercial or financial relationships that could be construed as a potential conflict of interest.

## Publisher's Note

All claims expressed in this article are solely those of the authors and do not necessarily represent those of their affiliated organizations, or those of the publisher, the editors and the reviewers. Any product that may be evaluated in this article, or claim that may be made by its manufacturer, is not guaranteed or endorsed by the publisher.
